# From Deformation Monitoring to Mechanism Insight: Assessing Sudden Subsidence Risk via an Improved 2D SBAS-InSAR and Physical Modeling Approach

**DOI:** 10.3390/s26020562

**Published:** 2026-01-14

**Authors:** Qiu Du, Guangli Guo, Huaizhan Li, Liangui Zhang, Fanzhen Meng, Zhenqi Hu, Jingchao Sun

**Affiliations:** 1 School of Environmental Science and Spatial Informatics, China University of Mining and Technology, Xuzhou 221116, China; duqiu@cumt.edu.cn (Q.D.); lihuaizhan@cumt.edu.cn (H.L.); huzq1963@cumt.edu.cn (Z.H.); sunjingchao@cumt.edu.cn (J.S.); 2 Department of Geological Survey, Yankuang Energy Group Co., Ltd., Jining 273500, China; ykzhangliangui@126.com (L.Z.); ykjtmfz@163.com (F.M.)

**Keywords:** mining subsidence, SBAS-InSAR, physical simulation, weakly cemented strata, risk assessment

## Abstract

**Highlights:**

**What are the main findings?**

- The study successfully developed and validated a refined 2D SBAS-InSAR monitoring technique. This method significantly mitigates the influence of north–south horizontal displacement, thereby reducing the comprehensive error in measuring the mining-induced subsidence basin from 50 mm to less than 20 mm, and allows for the precise determination of its boundary angles.
- Through a coupled analysis of remote sensing data and physical simulation results, the research quantitatively identified the critical mechanical conditions—specifically the width-to-depth ratios of the mining panel—that lead to the sequential rupture of key strata at different levels (0.21–0.27 for Yan’an Formation, 0.53–0.82 for Zhiluo Formation, and 1.22–1.34 for Zhidan Formation).

**What are the implications of the main findings?**

- The proposed technical framework and the quantified thresholds provide a concrete theoretical foundation for building a predictive early-warning system. This moves the field beyond post-event description towards proactive risk assessment of sudden subsidence in mining areas with similar geological conditions.
- The findings offer critical scientific guidance for safe and green mining practices globally. By understanding the mechanisms and preconditions of key stratum failure, mining strategies can be optimized to prevent catastrophic ground collapse, thereby enhancing operational safety and minimizing environmental impact.

**Abstract:**

Safe and efficient coal mining faces a global challenge in predicting sudden surface subsidence whose mechanisms remain unclear. This study, centered on deep coal seams in China’s Ordos Basin, examines the risk of abrupt subsidence controlled by high-positioned, ultra-thick, and weakly cemented key strata. We adopt an integrated “observation–experiment–model” paradigm. First, we construct a spatial decoupling model to analyze errors in 1D SBAS-InSAR monitoring, leading to a refined 2D method that reduces the three-dimensional monitoring error from 50 mm to under 20 mm. Based on this, the subsidence basin’s boundary angles are accurately determined as 52.3°–58.6° (strike) and 44.3°–48.2° (dip). Second, a large-scale physical simulation experiment visualizes the complete process of overburden failure up to the breaking of high-level key strata. Finally, by coupling remote sensing observations with experimental phenomena, a theoretical model is built to quantify the mechanical behavior of key strata, revealing the critical width-to-depth ratios for the rupture of the Yan’an Formation (0.21–0.27), Zhiluo Formation (0.53–0.82), and Zhidan Group (1.22–1.34). The research not only delineates surface subsidence morphology under special geological conditions but also answers the core questions of why subsidence occurs and when mutation may happen, thereby laying a theoretical foundation for a comprehensive early-warning model for mining areas worldwide.

## 1. Introduction

Coal resources, as a cornerstone of the global energy structure, are crucial for worldwide economic development, and their safe and efficient extraction is of paramount importance. However, underground coal mining, while meeting the energy demands of human society, profoundly alters the geological environment of mining areas, triggering widespread surface subsidence disasters [1]. This phenomenon is notably observed in major coal mining regions across the world, such as China’s Ordos Basin [2,3], Germany’s Ruhr region, and Poland’s Upper Silesia, constituting a transboundary common challenge in science and engineering. Surface subsidence manifests not only as large-scale, slow, and continuous settlement but also, more critically, exhibits potentially discontinuous and abrupt characteristics. When mining activities reach a certain scale, the instability of the overlying rock structure may induce sudden, large-scale collapses. The damage inflicted on surface structures, infrastructure (such as railways [4,5,6], highways [7,8,9], and pipelines [10,11,12]), and the natural ecological environment is catastrophic and extremely difficult to predict and issue warnings for. Consequently, from a global perspective, developing advanced monitoring technologies and theoretical models to accurately characterize the propagation process of surface subsidence and deeply reveal its mechanical mechanisms—particularly the transition from gradual deformation to sudden, catastrophic collapse—has become an international research frontier [13]. This research direction aims to promote the coordinated development of mining engineering and the geological environment and ensure a sustainable future for mining areas.

Over the past two decades, breakthroughs in space-based Earth observation have revolutionized the paradigm of surface deformation monitoring. Interferometric Synthetic Aperture Radar (InSAR) and its time-series techniques, such as Small Baseline Subset InSAR (SBAS-InSAR) [14,15], have been successfully applied to monitor various crustal movements, including earthquakes [16,17,18], volcanic activity [19,20,21], land subsidence, and mining-induced settlement, leveraging their advantages of broad coverage, high precision, and high spatial density. By selecting appropriate spatiotemporal baseline thresholds to combine interference pairs, SBAS-InSAR technology effectively mitigates decorrelation and atmospheric delay effects, achieving millimeter-level accuracy in capturing slow surface deformation processes.

However, it is crucial to recognize that surface deformation measurements obtained by conventional SBAS-InSAR are essentially “one-dimensional,” as the results represent algebraic projections of deformation along the satellite’s Line of Sight (LOS) [22,23]. This geometric ambiguity significantly hinders accurate interpretation of the true three-dimensional (3D) deformation patterns within a subsidence basin, particularly the vertical settlement and horizontal extension/compression, making it difficult to meet the requirements for refined, model-based research on subsidence mechanisms. To overcome this limitation, methods [24,25,26] for deriving two-dimensional (2D) and even 3D time-series (TS) InSAR solutions by integrating multi-platform (e.g., Sentinel-1, ALOS-2) and multi-geometry (ascending and descending orbits) SAR data have emerged. These approaches can decompose LOS deformation into vertical and east-west (or north–south) components, providing displacement fields that more closely represent the physical reality. This advancement offers unprecedented data support for progressing from simple deformation “observation” to in-depth mechanical mechanism “inversion”.

High-resolution surface deformation fields represent a crucial step in revealing mining-induced subsidence patterns, yet they remain, in essence, a form of “remote sensing” of the surface response to deep rock mass mechanical processes. The core scientific challenge in mining subsidence lies in understanding how stress disturbances induced by underground extraction cause hundreds of meters of overlying strata to deform, separate, fracture from the bottom up, and ultimately transmit the failure effects of the surrounding rock mass to the surface [27,28]. Within this complex chain of rock mass structural failure, the “key stratum” theory posits that one or several layers of hard, thick strata control the movement patterns and failure intervals of the entire overburden [29]. In the deep coal mining areas (mining depth > 500 m) of the central Ordos Basin, such as the Yingpanhao Coal Mine in Wushen Banner, Ordos City, with an average burial depth exceeding 700 m, the presence of these high-positioned, extremely thick key strata, along with specific geological conditions featuring thick, weakly cemented sandstone layers [30,31] and a thin surface layer of aeolian sand, often leads to a phased and discontinuous character of overburden failure [32]. This presents a significant difference compared to the overburden deformation and failure patterns observed in the mining areas of the eastern Chinese plains. When the continued fracturing of the immediate roof causes the suspended area of the high-positioned, thick key stratum [33] to reach its ultimate span, its instantaneous rupture can release enormous energy, highly likely manifesting on the surface as sudden, severe collapse or powerful dynamic disasters [34]. Consequently, the current frontier and challenge in mining subsidence research under similar geological conditions in the central Ordos Basin have shifted from “how to measure surface deformation more precisely” to “how to establish a quantitative, mechanistic link between surface deformation and the fracturing process of the overburden, particularly the key strata.” This necessitates a deep integration of advanced surface monitoring data with physical simulation and numerical/theoretical analysis tools capable of reproducing the failure processes within the deep rock mass.

Facing the challenges mentioned above, this study moves beyond singular observational or simulation approaches to adopt a systematic research paradigm driven by the triad of “observation–experimentation–modeling”. Taking the Yingpanhao Coal Mine, a typical high-intensity mining area in the central Ordos Basin, as a case study, we first utilized dense observational data from the European Space Agency’s (ESA) Sentinel-1 satellite constellation. Applying advanced two-dimensional (2D) SBAS-InSAR technology, we reconstructed the complete spatiotemporal evolution pattern of the ground surface subsidence field in the study area, precisely revealing the spatial characteristics and migration patterns of the subsidence basin. To visualize the overburden failure mechanisms behind the surface subsidence, we designed and conducted a large-scale physical similarity simulation experiment. This experiment can visually and dynamically reproduce the entire process from coal seam excavation to the fracture of key stratum and finally to surface subsidence, providing irreplaceable visual and quantitative evidence for understanding the temporal relationship of rock layer movement, the development law of delamination and the premonittive information of instability of key stratum. Ultimately, we coupled the results inverted from SAR satellite remote sensing with the phenomena observed in the physical simulation to construct a theoretical model capable of describing the mechanical behavior of the key stratum. Through this cross-validation and iterative feedback of multi-source information, this study aims to achieve the following core scientific objectives: not only to describe “how the surface subsidence manifests” under the specific overburden structure with high-positioned, ultra-thick, weakly cemented strata in the central Ordos Basin but, more importantly, to explain “why it occurs in such a manner” and predict “when abrupt subsidence might happen”. This work lays a solid theoretical foundation for establishing a risk early warning model for sudden surface collapse based on the integration of monitoring data and mechanical principles and offers a scientific framework with universal reference value for the safe and green mining in other mining areas with similar geological conditions worldwide.

The structure of the subsequent parts of this paper is as follows: Section 2 introduces the geographical location, stratigraphic structure, and coal mining status of the study area, detailing the 2D SBAS-InSAR method used and the SAR image data sources processed. Section 3 presents the 2D SBAS-InSAR results and their validation. Section 4 elaborates on the design of the similarity simulation experiment and its results, describing the internal deformation and failure characteristics of the overburden in the study area. Furthermore, based on rock mechanics theory, it provides an in-depth analysis of the intrinsic mechanisms of rock mass fracturing, which is cross-verified with the experimental results. Finally, Section 5 summarizes the paper and provides an outlook for future research directions.

## 2. Materials and Methods

### 2.1. The Geographical Location and Stratigraphy of the Study Area

**Figure 1:**
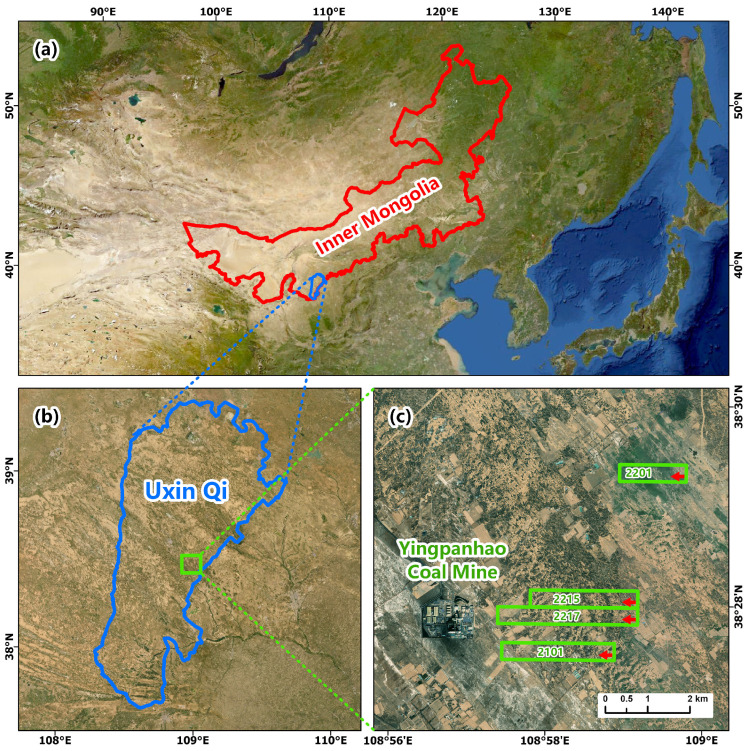
The geographical location of the study area. (**a**) Inner Mongolia. (**b**) Uxin Qi. (**c**) Yingpanhao Coal Mine.

As shown in Figure 1, the study area belongs to the temperate semi-arid continental climate. The terrain of the mining area is relatively flat. The landscape is mostly characterized by sand dunes, with the former predominating.

**Figure 2:**
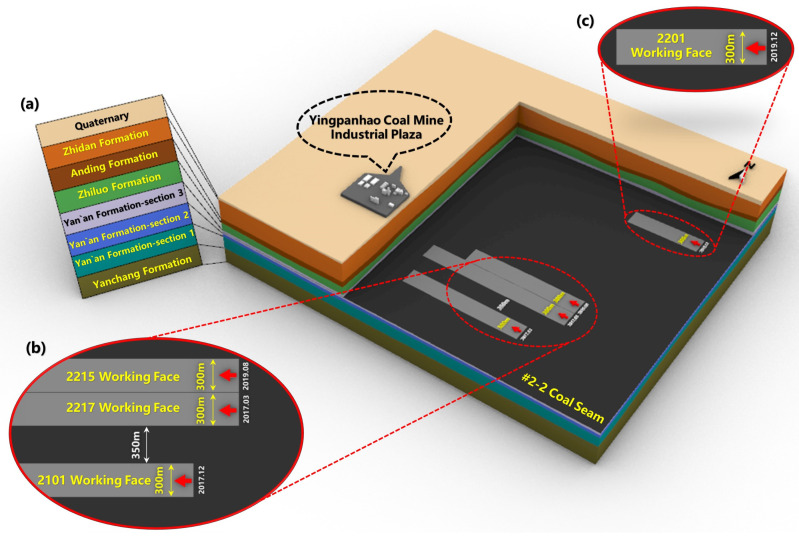
The stratigraphic structure of the study area. (**a**). Stratigraphic note; (**b**,**c**). Distribution diagrams of the working faces 2201, 2101, 2215, and 2217.

The stratigraphy of the study area is shown in Figure 2. The Ordos Plateau syncline, in which the study area is located, is a relatively complete and stable tectonic unit, with no discontinuous structural surfaces such as large faults. This indicates that the integrity of the rock formations in the study area is fairly good. The thickness of Zhiluo Formation and its overlying strata is large, which generally exceeds 100 m. The average thickness of the sandstone layer of the Zhidan Group of the Lower Cretaceous reaches 341.33 m. The buried depth of No.2-2 coal seam currently mined in Yingpanhao Coal Mine is 660.38–783.68 m, with an average of 722.88 m, an average dip angle of 1°, a thickness of 5.21–7.33 m, and an average thickness of about 6.41 m. There are four working faces in Yingpanhao Coal Mine, which are 2101, 2217, 2215, and 2201 working faces, as shown in Figure 2b,c. These four working faces are all arranged as strike longwall comprehensive mechanized mining faces with a mining width of 300 m, a mining thickness of 5.5 m and a total design advance length of more than 2 km. The roof is managed by caving method.

### 2.2. The Principle of 2D SBAS-InSAR Technology

This section analyzes the spatial geometric relationship between the surface displacement observed by one-dimensional (1D) SBAS-InSAR and the actual surface subsidence, establishes a mathematical model between the surface subsidence observation and the incident angle and measurement point position of the SAR signal, and further proposes the basic principle of 2D SBAS-InSAR.

#### 2.2.1. The Geometric Relationship Between the Line-of-Sight (LOS) Displacement Observations of 1D SBAS-InSAR and the Actual Surface Displacement (ASD)

**Figure 3:**
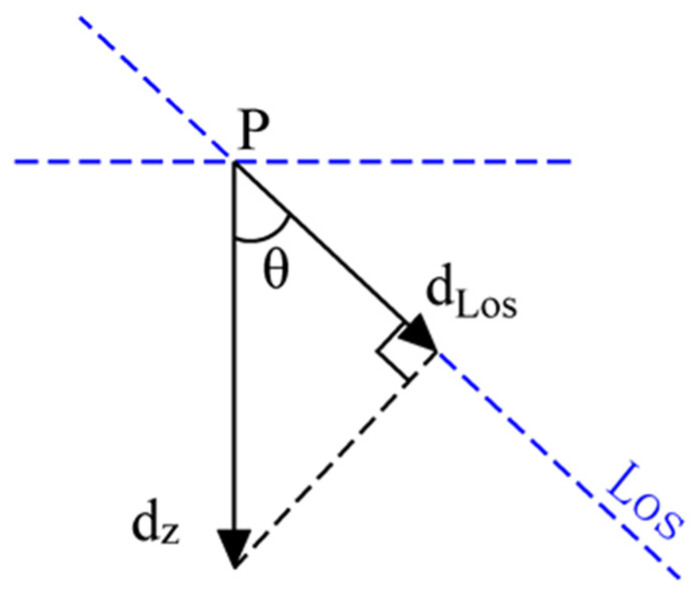
The relationship between surface vertical displacement and LOS displacement.

According to the principle of SAR side-looking imaging, InSAR and its derived time series SAR technology can only measure the projection of the real surface displacement vector of the target area on the LOS of the radar signal when using single-track SAR image data. The existing InSAR data processing software (like SARscape v5.7.0) usually assumes that the surface only has vertical displacement (VD). Under this assumption, the VD and the LOS displacement of the surface deformation zone have the cosine corner geometric relationship shown in Figure 3.

Under this geometric relationship, the relationship between the VD of the surface $d_{Z}$, the LOS displacement $d_{Los}$, and the incident angle of the radar signal θ can be expressed as shown in Equation (1). Based on this Equation, $d_{Z}$ can be calculated by $d_{Los}$ and θ.

(1) $$d_{z}=\frac{d_{Los}}{\cos\theta }$$

However, a large number of measured data and theoretical analysis show that the direction of surface displacement caused by underground coal resource mining points to the center of goaf and contains both vertical and horizontal displacement components, which has typical 3D displacement characteristics.

**Figure 4:**
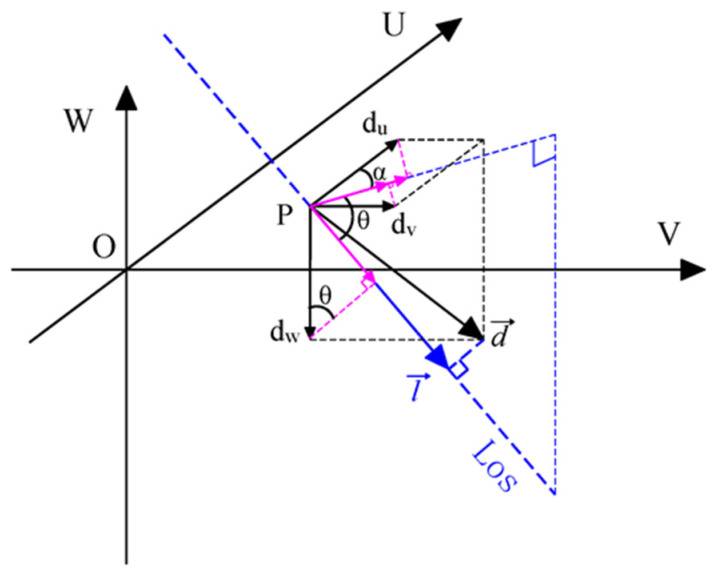
Spatial relationship between ASD and radar LOS deformation in mining area.

Figure 4 shows the spatial geometric relationship between the 3D displacement of a certain point on the surface of the mining area and the projection in the LOS direction of the radar signal. The displacement of the point on the surface of the mining area can be expressed as a vector $\vec{d}=\left(du,dv,dw\right)$, and the unit vector in the LOS direction of the radar signal can be expressed as $\vec{l}=\left(du,dv,dw\right)$. Among them, du, dv, and dw are, respectively, the east–west component, north–south component, and vertical component of the displacement of a point on the surface; θ and α represent the incident angle and azimuth of the radar signal, respectively. From the projection relation of one vector to another vector in three-dimensional space, the projection vector $\vec{d_{Los}}$ of the real displacement vector $\vec{d}$ of each point on the surface in the direction along the LOS can be obtained:

(2) $$\begin{array}{cc} \vec{d_{Los}} & =\frac{\vec{d}\cdot \vec{l}}{{\left|\vec{l}\right|}^{2}}\times \vec{l}\\ & =\left(du\sin\theta \sin\alpha +dv\sin\theta \cos\alpha -dw\cos\theta \right)\times \vec{l} \end{array}$$

Since $\vec{l}$ is a unit vector, the absolute value of the LOS displacement of the point is:

(3) $$\left|\vec{l}\right|=du\sin\theta \sin\alpha +dv\sin\theta \cos\alpha -dw\cos\theta$$

Taking Equation (3) into Equation (1), the VD of a point on the surface of the mining area measured by SARScape and other commonly used software can be obtained:

(4) $$\begin{array}{cc} ∆z & =\frac{-\left|\vec{d_{Los}}\right|}{\cos\theta }\\ & =\frac{dw\cos\theta -du\sin\theta \sin\alpha -dv\sin\theta \cos\alpha }{\cos\theta }\\ & =dw-du\tan\theta \sin\alpha -dv\tan\theta \cos\alpha \end{array}$$

In Equation (4), the negative sign in the first row of molecules indicates that the preset displacement direction is downward, opposite to the coordinate axis direction shown in Figure 4. By swapping the last line of Equation (4) to the left of the equal sign, we can obtain:

(5) ∆z−dw=−dutanθsinα−dvtanθcosα

Equation (5) is the systematic error calculation formula of the VD of each point on the surface of the mining area. From this equation, it can be seen that in the case of horizontal and vertical component displacement on the surface of the mining area, the vertical displacement of the surface obtained by SBAS-InSAR and other time series InSAR methods based on monorail SAR image data is not only composed of the actual VD of the surface, but also the horizontal east–west component and north–south component of the surface displacement, as well as the influence of the incident angle θ and azimuth angle α of the radar signal.

The incident angles θ of the ascending SAR images on the east and west sides of the Sentinel-1 A satellite used in this study are $\theta_{E}=34.0{}^{\circ}$ and $\theta_{W}=44.5{}^{\circ}$, and the azimuth angles α are $\alpha_{E}=79.3{}^{\circ}$ and $\alpha_{W}=80.6{}^{\circ}$, respectively. Then, $\tan\theta_{E}=0.6745$, $\tan\theta_{W}=0.9827$, $\sin\alpha_{E}=0.9826$, $\sin\alpha_{W}=0.9865$, $\cos\alpha_{E}=0.1857$, $\cos\alpha_{W}=0.1633$. From this, the coefficient values in Equation (5) can be obtained:

(6) $$\left\{\begin{array}{c} \begin{array}{c} \tan\theta_{E}\sin\alpha_{E}\approx 0.6628\\ \tan\theta_{W}\sin\alpha_{W}\approx 0.9694 \end{array}\\ \begin{array}{c} \tan\theta_{E}\cos\alpha_{E}\approx 0.1253\\ \tan\theta_{E}\cos\alpha_{W}\approx 0.1605 \end{array} \end{array}\right.$$

From Equation (6), it can be seen that the value ranges of both $\tan\theta_{E}\sin\alpha_{E}$ and $\tan\theta_{W}\sin\alpha_{W}$ are between (0,1). Among them, the values of coefficient tanθsinα are all greater than 0.5, even close to 1, while the values of coefficient tanθcosα are all close to 0.1. This indicates that term dvtanθcosα in Equation (5) can actually be ignored. Then Equation (5) can be simplified as:

(7) ∆z−dw=−dutanθsinα

The above Equation (5) indicates that there is a systematic difference between the surface settlement values measured by the traditional 1D SBAS-InSAR and the actual surface settlement values. This difference is mainly affected by the horizontal displacement of the surface and the incident angle of the SAR signal.

#### 2.2.2. The Basic Principle of 2D SBAS-InSAR

According to the above analysis, it is difficult to obtain accurate surface subsidence value by 1D SBAS-InSAR. To this end, we propose 2D SBAS-InSAR method to effectively reduce the systematic difference mentioned above.

The ASD of a point on the surface is regarded as an unknown vector $\vec{d}=\left(du,dv,dw\right)$, then Equation (4) can be regarded as a linear equation containing three unknown displacement components du, dv, and dw. The VD obtained by SBAS-InSAR processing and the incident angle θ and azimuth angle α of the satellite radar signal are known components, which constitute the coefficients of the unknown components. According to the knowledge of linear algebra, it is necessary to establish at least 3 equations with different coefficients to solve the linear equation with 3 unknown components. It can also be understood that SAR imaging at least 3 different angles should be carried out in the study area within a relatively short time interval.

However, most of the current SAR satellites use polar orbit side-looking mode for imaging. The radar signal LOS is basically close to the east–west direction plane. And it is difficult to form a non-coplanar observation condition. Therefore, it is impossible to directly solve the 3D surface deformation under the observation conditions of the current SAR satellite. Some researchers [24] have established the relationship between the vertical component and horizontal component of surface displacement by introducing the mathematical relationship between the surface inclination and horizontal displacement. They replaced du and dv in Equation (4) with expressions containing dw to solve the 3D deformation of the surface in the mining area. This method is essentially a kind of substitution method based on prior knowledge, which is suitable for the eastern China mining area that has basically mastered the law of surface movement and deformation. However, the research on the law of mining-induced surface movement and deformation in Ordos mining area is still in its infancy. Under the condition of not accumulating a large amount of measured data of surface deformation, it is impossible to determine the relationship between inclination deformation and horizontal displacement of each point on the surface above the goaf. Therefore, it is difficult to solve the three-dimensional deformation of the surface using such methods. Therefore, we did not use this method to obtain the 3D deformation of the surface.

The azimuth α of the radar signals from the SAR satellites that have been launched is close to 90° (or 270°). This indicates that the terms with a in Equation (5) approach 0, reducing the contribution of the north–south displacement components of the goaf surface to the VD system error $\left(∆z-dw\right)$. Therefore, Equation (7) containing only two components, du and dw, can be obtained. At this point, it is only necessary to establish a system of equations with two equations to solve it. That is to say, only two observations in different directions are needed to solve for the VD component and the east–west displacement component at a certain point on the surface above the goaf.

In our study, SAR image data from two adjacent orbits of Sentinel-1 A were used, and the incident angles of SAR signals on both sides of the orbit are different. Therefore, it can be considered as two different angles of observation. The dw on the left side of Equation (7) is moved back to the right side, and the VD of a point is calculated as $∆Z_{E}$ and $∆Z_{W}$ by SBAS on the east and west sides, respectively. Then the linear equations shown in Equation (8) can be established. Based on Equation (8), the VD component dw of surface displacement and the horizontal displacement component du in the east–west direction can be solved.

(8) $$\left\{\begin{array}{c} ∆Z_{E}=dw-du\tan\theta_{E}\sin\alpha_{E}\\ ∆Z_{W}=dw-du\tan\theta_{W}\sin\alpha_{W} \end{array}\right.$$

### 2.3. SAR Image Data Source and Auxiliary Data Source

We utilized SAR image data in IW mode SLC format from Sentinel-1 A satellite, which was freely distributed by the ESA. The Sentinel-1 A satellite has been continuously scanning and imaging the study area since 10 March 2017. The yellow area in the diagram shows the range of the Yingpanhao coal mine field. This range is located in the overlapping area of the western imaging area represented by the blue rectangular frame (imaging track Path84, number Frame120) and the eastern imaging area represented by the red rectangular frame (imaging track Path11, number Frame121). The main parameters of SAR images used are shown in the Table 1.

In order to avoid the error caused by the conversion of the elevation system in the SBAS-InSAR processing process, and to be consistent with the principle of InSAR interferometry to remove the terrain phase, we used the TanDEM-X DEM with a geodetic ellipsoid height, a reference ellipsoid WGS-84, and a spatial resolution of 90 m for auxiliary processing.

**Figure 5:**
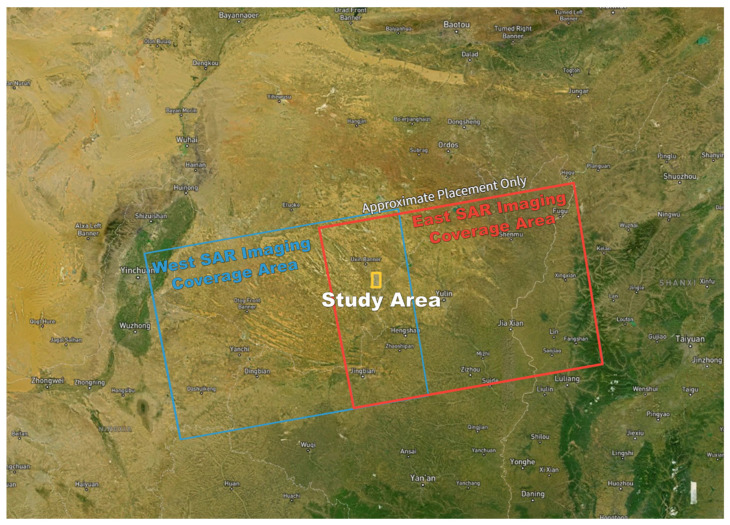
Sentinel-1 A SAR Imaging Coverage Area.

**Figure 6:**
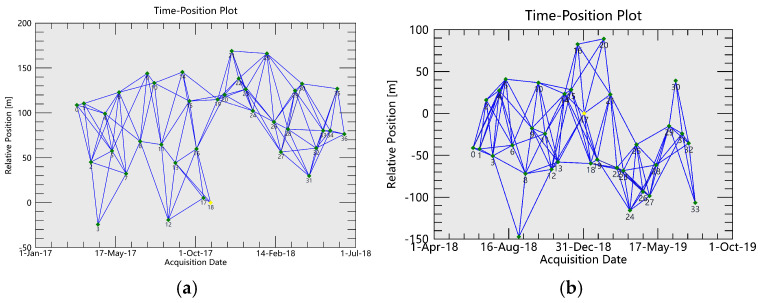

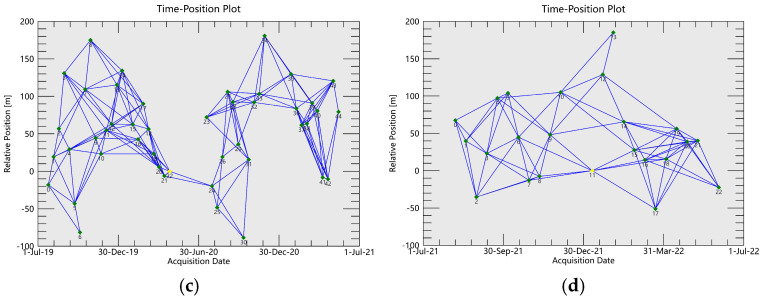
Spatio-temporal baseline connection diagrams of each segment (east SAR images). (**a**) First Period. (**b**) Second Period. (**c**) Third Period. (**d**) Fourth Period.

**Figure 7:**
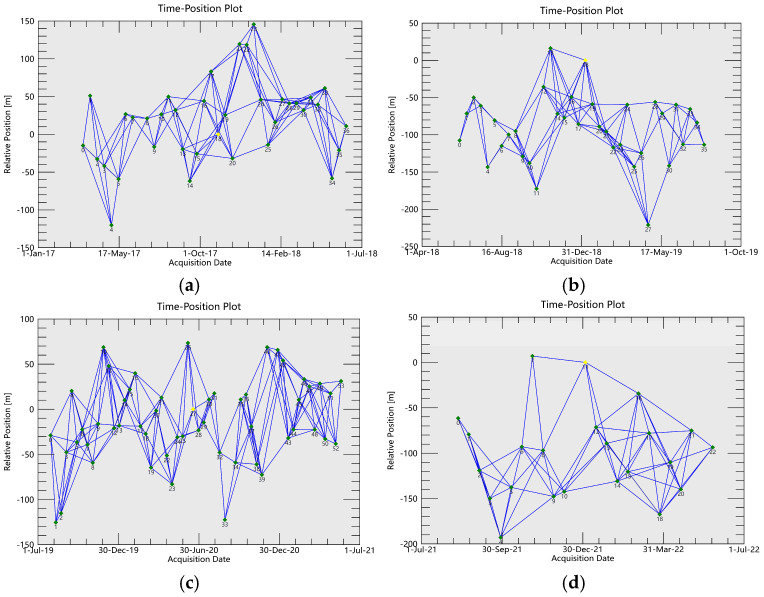
Spatio-temporal baseline connection diagrams of each segment (west SAR images). (**a**) First Period. (**b**) Second Period. (**c**) Third Period. (**d**) Fourth Period.

The total time span of this monitoring is longer than 5 years. In order to reduce the time-space decoherence interference, improve the data processing effect, and shorten the data processing time, this study combines the mining status of 2217, 2101, 2215 and 2201 four working faces in different time periods, and divides the east and west sides of the track SAR image set shown in Figure 5 into four periods according to the time sequence. The specific segmentation information is shown in Table 2. Figure 6 and Figure 7 are the spatio-temporal baseline connection maps of the SAR images on the east and west sides in 4 periods, respectively.

## 3. Results

In this section, we present the key monitoring outcomes and derived deformation characteristics obtained from both 1D and improved 2D SBAS-InSAR. And we summarize the surface deformation characteristics extracted from improved 2D SBAS-InSAR. The results depict the spatial–temporal evolution of subsidence basins under different mining stages and provide refined boundary parameters essential for interpreting subsequent deformation mechanisms.

### 3.1. The Processing Result of 1zD SBAS-InSAR

#### 3.1.1. Original Result of Surface Subsidence

**Figure 8:**
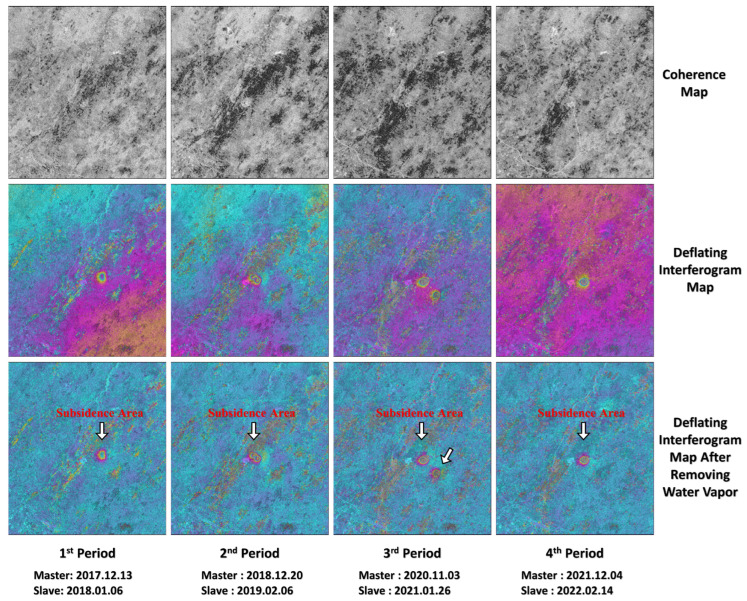
The interference process diagrams of typical image pairs in each periods (east).

**Figure 9:**
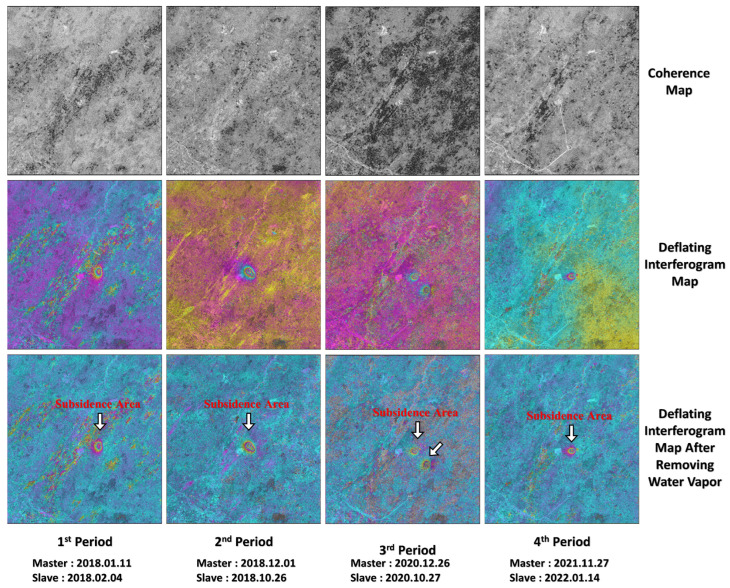
The interference process diagrams of typical image pairs in each period (west).

Figure 8 and Figure 9 are the coherence maps of partial image pairs of SAR images on the east and west sides in four periods, the interferograms after removing the ground effect and the interferograms after removing the atmospheric effect by using GACOS water vapor data, respectively. It can be seen from the figures that the coherence of SAR images at each time period on the east and west sides is good, and the deformation interference fringes are clear.

**Figure 10:**
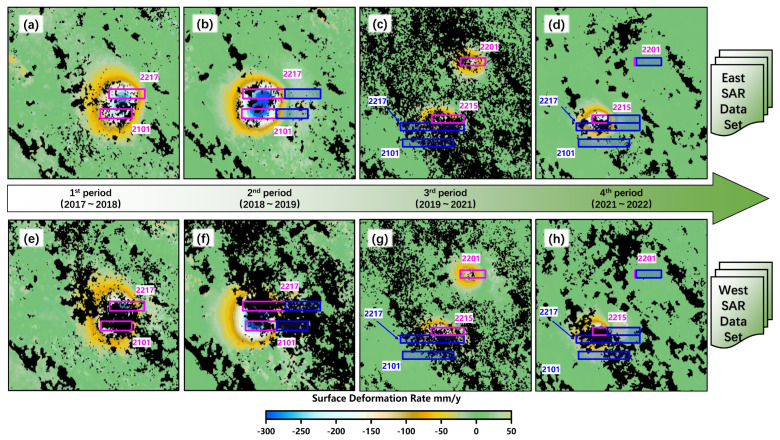
The surface deformation rate maps of Yingpanhao coal mine obtained by SBAS-InSAR. (**a**–**d**) The surface deformation rate maps made by East SAR data Set. (**e**–**h**) The surface deformation rate maps made by West SAR data Set.

Figure 10 shows the distribution map of the annual surface subsidence rate of Yingpanhao Coal Mine during the aforementioned four time periods.

As can be seen from Figure 10a,e, during the first period (2017–2018), an overall moving basin emerged on the surface after the mining of the 2217 and 2101 working faces. The calculation of the eastern orbital SAR image set shows that the maximum annual subsidence rate of the surface in this subsidence basin is 308.87 mm/y. The calculation of the SAR image set of the west orbit shows that the maximum annual subsidence rate of the surface of this subsidence basin is 308.94 mm/y.

As can be seen from Figure 10b,f, during the second period (2018–2019), the planar position of the surface moving basin shifted along the direction of the mining progress of the 2217 and 2101 working faces. The calculation of the eastern orbital SAR image set shows that the maximum annual subsidence rate of the surface in this subsidence basin is 356.57 mm/y. The calculation of the SAR image set of the west orbit shows that the maximum annual subsidence rate of the surface of this subsidence basin is 353.35 mm/y.

As can be seen from Figure 10c,g, during the third period (2019–2021), a separate mobile basin was formed on the surface above the two working faces 2215 and 2201, respectively. After statistics, the SAR image sets on the east and west sides calculated that the maximum annual subsidence rates of the surface above the 2215 working face were 277.74 mm/y and 289.67 mm/y, respectively, while those above the 2201 working face were 144.84 mm/y and 145.83 mm/y, respectively.

As can be seen from Figure 10d,h, during the fourth period (2021–2022), the maximum annual subsidence rates of the surface above the 2217 working face were 302.57 mm/y and 296.60 mm/y, respectively. The maximum annual subsidence rates of the surface above the 2201 working face are only 8.68 mm/y and 18.54 mm/y, respectively.

#### 3.1.2. Comparison of the 1D SBAS-InSAR with the Level Monitoring

The following compares the 1D SBAB-InSAR surface subsidence data with the leveling subsidence data to verify the systematic deviation of 1D SBAS-InSAR. We take the first period (March 2017 to June 2018) as an example to compare the surface subsidence curve obtained by the SBAS on the east and west sides and the subsidence time series curve of the special points.

**Figure 11:**
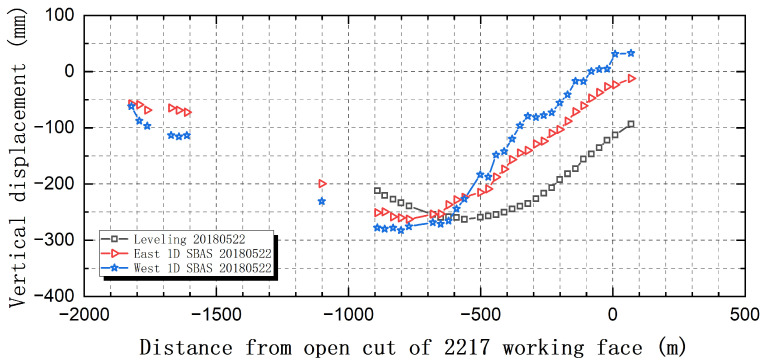
Comparison of 1D SBAS-InSAR and Leveling Ground Subsidence Curves.

**Figure 12:**
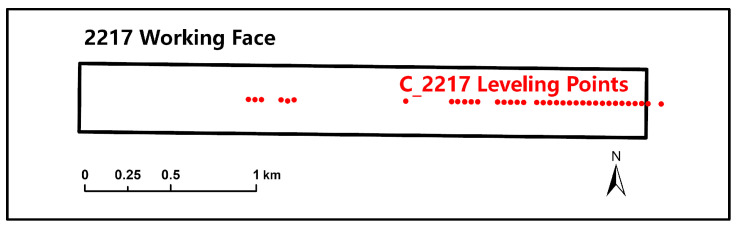
The observation lines for ground movement above 2217 and 2101 working faces.

The last leveling observation date in the first period is 22 May 2018. On the same day, L_2217_ was 1128.9 m. Figure 11 shows the comparison results of the leveling quasi-subsidence curves along the leveling observation line C_2217_ (Figure 12) and the 1D SBAS-InSAR subsidence curves on the east and west sides. As shown in Figure 11, there are obvious differences in the surface subsidence curves of the three survey lines. This indicates that there is indeed a systematic deviation between the surface subsidence obtained directly by 1D SBAS-InSAR processing and the actual surface subsidence.

### 3.2. The Processing Result of 2-D SBAS-InSAR

**Figure 13:**
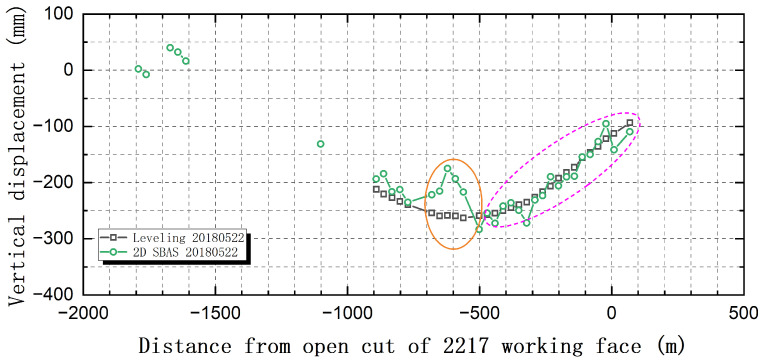
Comparison of SBAS-InSAR and Leveling Ground Subsidence Curves.

Figure 13 shows the comparison between the corrected surface subsidence curve of SBAS-InSAR by 2D SBAS-InSAR and the leveling subsidence curve of the observation line C_2217_ at the end of the first time period (22 May 2018). It can be seen from each subgraph that after correction, the 2D SBAS-InSAR surface subsidence curve is basically consistent with the leveling surface subsidence curve, especially in the edge area of the surface subsidence basin (as shown in the purple oval dotted line range in Figure 13). The correction effect of the central area of the surface subsidence basin (as shown in the yellow oval dotted line range in Figure 13) is slightly worse than that of the marginal area of the subsidence basin.

Based on the fundamental principles of SBAS-InSAR technology, the primary causes of this phenomenon can be attributed to the following two factors.

First, in the central region of the subsidence basin, the displacement gradient is relatively high, resulting in reduced coherence of the interferometric image pairs. Consequently, the line-of-sight displacement derived from such data inherently contains a significant degree of random error.

Second, on the western side of the subsidence basin’s center, the line-of-sight direction of radar signals obtained from both ascending and descending SAR acquisitions aligns closely with the actual surface displacement direction in that area. This alignment leads to minimal discrepancies between the SBAS-derived subsidence measurements from the ascending and descending tracks, making the observed deformation comparable to the intrinsic measurement uncertainty of the SBAS-InSAR technique. According to the principle of error propagation, when the magnitude of actual ground deformation approaches the level of measurement error, the vertical displacement estimated through inversion using Equation (8) may exhibit a non-negligible deviation from the true displacement value.

Further analysis of the difference between the 2D SBAS-InSAR and the leveling result in Figure 13 shows that the observation error of 2D SBAS-InSAR at the edge of the surface subsidence basin is within 20 mm, and the maximum error of observation in the middle of the basin is not more than 50 mm. Under this accuracy, we use 2D SBAS-InSAR to extract and analyze the boundary characteristics of the surface subsidence basin in the study area.

Yang et al. [35] proposed a method to adaptively evaluate the overall accuracy of InSAR subsidence estimation by monitoring values in non-deformation areas and delineate the boundary of mining-induced surface movement basins based on the confidence interval. Based on this method, this study assumes that the relatively high-precision leveling subsidence data are consistent with the actual subsidence of the surface, estimates the standard deviation (STD) of the SBAS subsidence after correction in the edge area of the moving basin, and then obtains the boundary of the mining surface movement basin with 1.65 times the standard deviation and a confidence interval of 90%. After calculation, the STD value of the modified SBAS in the edge area of the surface movement basin in this study area is 27.4 mm. In order to obtain the moving basin boundary with a confidence interval of 90%, 45.2 mm can be obtained based on the obtained STD value multiplied by 1.65 times. In order to facilitate the statistical drawing, this study appropriately increases the SDT multiple, and the threshold for the actual delineation of the boundary of the surface movement basin is set to sink 50 mm. The confidence interval of the surface movement basin boundary is theoretically better than 90%. Based on this, the characteristics of surface movement basin boundary under different mining conditions in Yingpanhao Coal Mine are analyzed.

#### 3.2.1. Boundary Characteristics Under Insufficient Single-Face Mining

**Figure 14:**
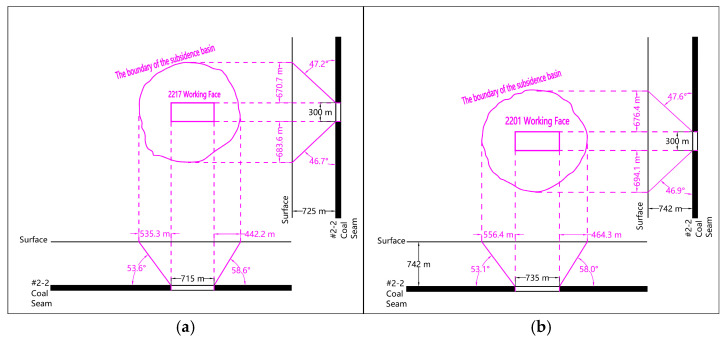
Ground subsidence range maps for single working face mining. (**a**) 2217 working face; (**b**) 2201 working face.

Figure 14 is the analysis diagram of the boundary characteristics of the surface movement basin when the mining lengths of the 2217 and 2201 working faces are 715 m and 735 m, respectively, obtained by the 2D SBAS-InSAR method. As shown in Figure 14, under the condition of extremely insufficient mining of single working face corresponding to the initial mining stage of 2217 and 2201 working faces, the range of boundary distance in front of the working face is 535.3 m~556.4 m, and the range of boundary angle in front of the working face is 53.1°~53.6°. The range of the rear boundary distance is 442.2 m~464.3 m, and the range of the rear boundary angle is 58.0°~58.6°. The range of dip boundary distance is 670.7 m~694.1 m, and the range of dip comprehensive boundary angle is 46.7°~47.6°.

#### 3.2.2. Boundary Characteristics Under Interval Mining of Section Coal Pillars

**Figure 15:**
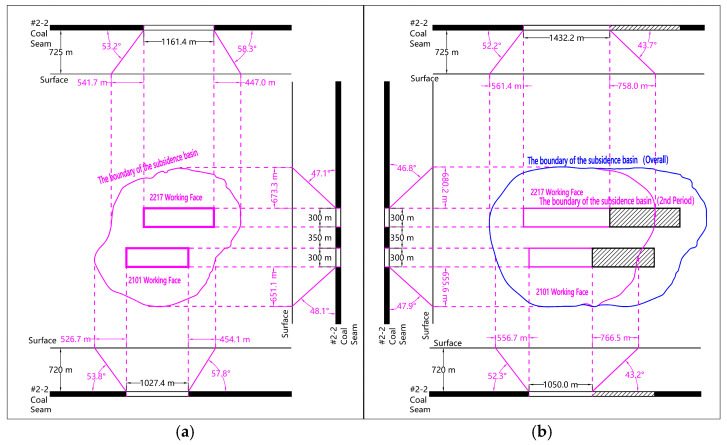
Ground subsidence range maps for mining under the interval of section coal pillar. (**a**) First Period; (**b**) Second Period.

The total process of simultaneous mining of the two working faces of 2217 and 2101 crosses the first two time periods of the designed SBAS-InSAR data processing. Therefore, the boundary characteristics of the surface movement basin under the condition of interval mining of section coal pillars are analyzed by superposition of the boundary of the surface mining subsidence basin at the end of the first and second time periods. As shown in Figure 15, under the condition of interval mining of section coal pillars, the range of forward boundary distance of mining area is 526.7 m ~ 561.4 m, and the range of forward boundary angle is 52.2°~53.8°. The range of the rear boundary distance is 447.0 m~454.1 m, and the range of the rear boundary angle is 57.8°~58.3°. The range of dip boundary distance is 651.1 m~680.2 m, and the range of dip comprehensive boundary angle is 46.8°~48.1°.

#### 3.2.3. Boundary Characteristics Under Continuous Mining of Adjacent Working Faces

**Figure 16:**
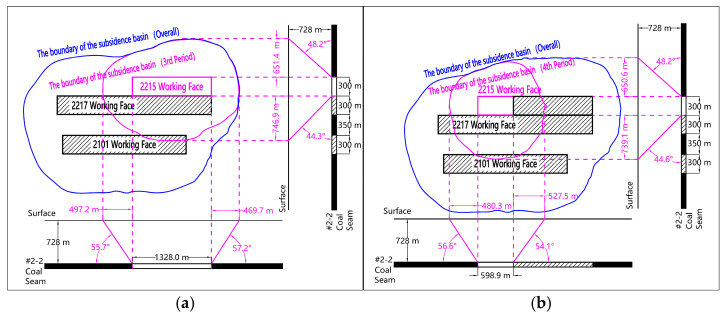
Ground subsidence range maps for continuous mining in double adjacent working faces. (**a**) Third Period; (**b**) Fourth Period.

The total mining process of 2215 working faces spans the last two SBAS-InSAR data processing segments designed in this study. Based on the superposition of the surface subsidence data at the end of the third and fourth time periods, the boundary characteristics of the surface movement basin under the mining state of the adjacent working faces of the double working faces can be analyzed. As shown in Figure 16, under the condition of adjacent mining of double working faces, the range of forward boundary distance of new local surface movement basin is 480.3 m~497.2 m, and the range of forward boundary angle is 55.7°~56.6°. The range of the rear boundary distance is 469.7 m~527.5 m, and the range of the rear boundary angle is 54.1°~57.2°. The lateral tendency boundary distance of the solid coal pillar ranges from 650.6 m to 651.4 m, and the tendency comprehensive boundary angle is 48.2°. The range of lateral inclination boundary distance of goaf is 739.1 m~746.9 m, and the comprehensive inclination boundary angle is 44.3°~44.6°.

The boundary angle of the surface subsidence basin under the above three types of mining conditions shows the following characteristics: (1) The rear boundary angle of the strike direction is obviously larger than dip comprehensive boundary angle. (2) The boundary angle of the subsidence basin in the eastern mining area of China is about 50°–60°, and the dip boundary angle of the subsidence basin in the study area is obviously small (44.3°–48.2°). This shows that the surface subsidence caused by underground coal resources mining in the study area is wider.

## 4. Discussion

This section synthesizes the monitoring results, physical simulation phenomena, and mechanical modeling to interpret the mechanisms driving the observed deformation patterns. Through this integration, the underlying causes of key-stratum failure and sudden subsidence are clarified.

### 4.1. Physical Simulation of Overburden Failure

#### 4.1.1. Similarity Model Design and Experimental Procedure

Similar material simulation is an experimental method based on the principle of similarity. This method is widely used in hydraulic engineering, construction engineering, mining engineering, and aerospace engineering. In this study, the two-dimensional planar stress similarity simulation experimental platform of the Mining Subsidence Research Institute of China University of Mining and Technology was used to simulate the characteristics of overlying strata movement in the study area. The experimental platform is 3.0 m long and 0.3 m wide. The height of the model can be freely adjusted according to the needs of the experiment, up to 1.5 m. The platform is equipped with an indoor constant temperature and humidity regulator, which can greatly reduce the disturbance of environmental factors such as temperature and humidity. The main steps of similar material simulation experiments are determining the model similarity ratio, selecting similar materials with ratios, and designing a simulated mining scheme.

(1) Determining the Model Similarity Ratio

Geometric similarity, motion similarity, and dynamic similarity are mainly considered in the study of mining overburden movement characteristics. There are 13 physical quantities contained in the 3 similarity conditions, which are Stratum Geometry l, Displacement of Rock-soil Mass d, Stress σ, Strain ε, Physical Mechanics Parameters of Stratum (Density ρ, Elastic Modulus E, Poisson Ratio υ, Compressive Strength $\sigma_{C}$, Tensile Strength $\sigma_{m}$, Internal Friction Angle φ, Cohesion φ), Time τ, Acceleration of Gravity g. Considering the test conditions, $C_{g}=1$, $C_{\rho }=1:1.6,$ and $C_{l}=1:500$. Then, the remaining 7 similarity ratios are:

Time similarity ratio: $C_{\tau }=\sqrt{C_{l}}=1:22.36$;

Mechanical properties similarity ratio: $C_{E}=C_{\sigma_{C}}=C_{\sigma_{m}}=C_{C}=C_{l}\cdot C_{\rho }=1:800$;

Displacement similarity ratio: $C_{d}=C_{l}=1:500$;

Stress similarity ratio: $C_{\sigma }=C_{l}\cdot C_{\rho }\cdot C_{g}=1:800$.

It should be noted that when the geometric similarity ratio $C_{l}$ is 1:500, the total height of the model should be 1.56 m according to the average mining depth of the prototype 740 m and the thickness of the coal seam floor of 40 m. This height has exceeded the maximum height of the model frame. In addition, the top aeolian sands in the stratigraphy of the study area are highly migratory and difficult to stabilize in a two-dimensional similarity model. In order to ensure the success of the simulation experiment, this study adopts removing the aeolian sands formation and part of the Zhidan Formation and replacing them with an equivalent load. The remaining thickness of the Zhidan Formation in the prototype after the operation is 185 m. The total height of the model is 1.026 m.

(2) Selecting Similar Materials with Ratios

**Figure 17:**
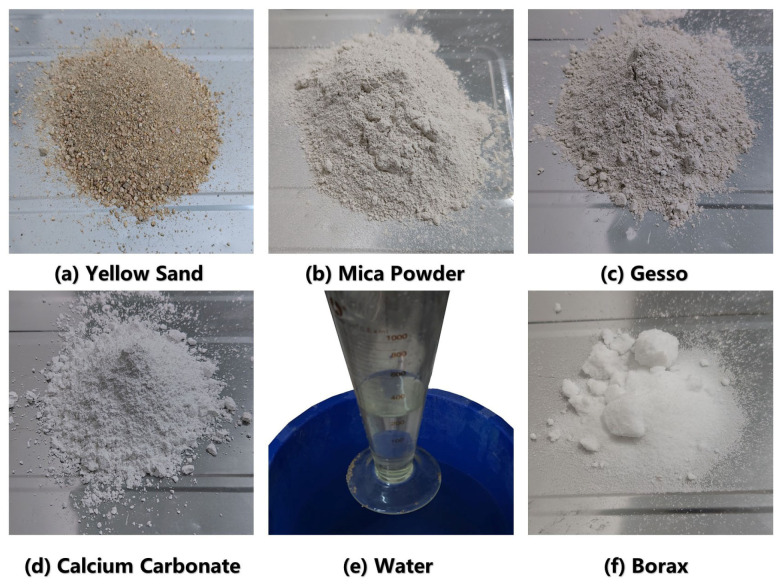
Similar materials.

Similar materials should be selected as far as possible to meet the requirements of stable mechanical properties, physical and mechanical properties closer to the prototype, easy to obtain, low cost, convenient processing. As shown in Figure 17, this study used yellow sand as aggregate, a mixture of gypsum and calcium carbonate as cement, and mica powder as an ingredient to enhance the plasticity of the material and to regulate the specific gravity of similar mixtures. In addition, pure water mixed with appropriate amount of borax (retarding effect) was used to fully mix and shape all similar materials.

According to the mechanical parameters of the prototype formation and the determined similarity ratios, materials mixing experiments can be carried out in order to set the similar weight ratios of materials for different formations. The physico-mechanical parameters and the weight ratios of the similar materials in this study are shown in Table 3 and Table 4, respectively.

(3) Building the Model and Designing Simulated Mining Scheme

**Figure 18:**
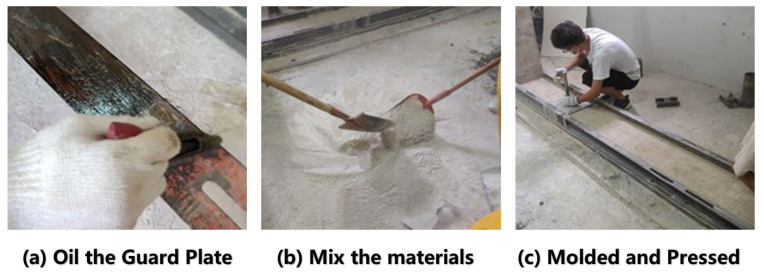
Part of the process of building a similar model.

**Figure 19:**
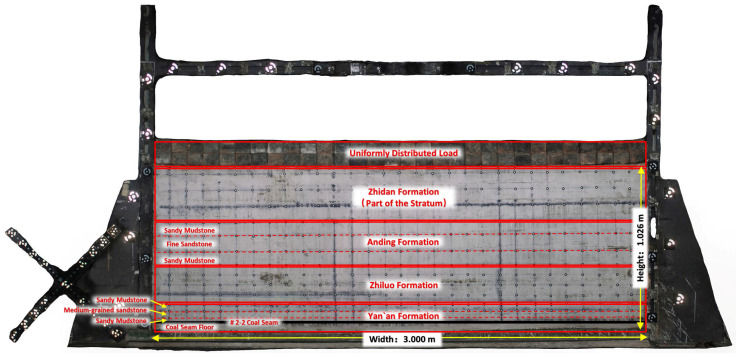
The completed similar material model.

The construction process of similar material model includes the following steps (Figure 18):

1. Clean model frames, guards, and the ingredient site to prevent debris from influencing and to ensure that the experimental environment is safe.
2. Oil the guards plate evenly to prevent them from sticking to similar model. Lifting equipment and bolts must be used to tighten the guard plate to the model frame.
3. In accordance with the designed similar ratio, the aggregate and cementing materials are evenly mixed and then poured into the pure water added by the appropriate amount of retarder to make it fully mixed.
4. The mixed material is poured evenly into the grooves between the guards, flattened and compacted properly.
5. After finishing the construction of the model (see Figure 19), it is let stand and stabilize under constant temperature and humidity for 7 to 10 days.

The 2D similarity model belongs to the plane stress model. Therefore, it is necessary to design a reasonable simulation mining scheme according to the actual situation. The simulation experiment scheme of this study can be divided into two parts.

First is single working face mining. It is simulated mining of coal seams within 600 mm width of the model at 50 mm intervals. Each simulated excavation is left to act for 12 h to allow the overburden rock to be fully deformed and destroyed. Then, according to the experimental results, the characteristics of the overburden rock movement in the strike profile are analyzed for the actual mining length of 25 m, mining width of 300 m, and the total length of the push mining within 300 m of the single working face.

Second is the continuous mining of adjacent multi-working faces. After the previous simulated mining is finished, the simulation is continued to excavate two working faces with a width of 600 mm. For avoiding the strong disturbance to the model caused by too fast excavation speed, the remaining two working faces are both excavated in six stages. In other words, the width of each excavation is 100 mm. The model is let stand for 2 h and then continues to excavate until the current working face is excavated. In addition, in order to ensure the sufficient deformation and failure, all of them are left to stand for 48 h after the completion of the simulated excavation.

#### 4.1.2. Overburden Failure Characteristics Under Different Mining Widths

(1) Single Working Face Mining

**Figure 20:**
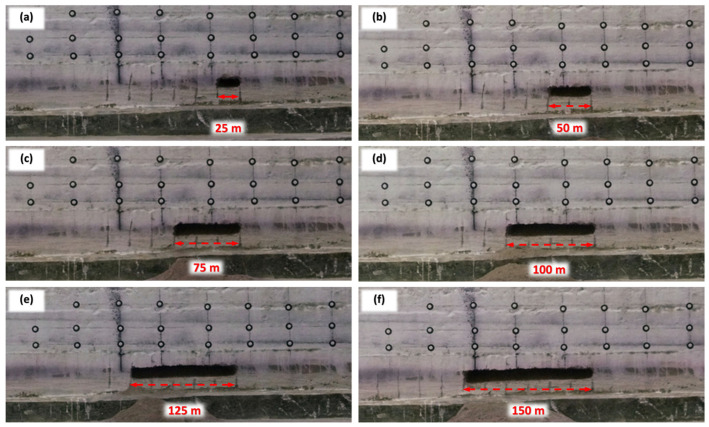
Overburden movement under simulated mining of 25 to 150 mm widths. (**a**) 25 m. (**b**) 50 m. (**c**) 75m.(**d**) 100m. (**e**) 125 m. (**f**) 150 m.

Figure 20 shows the process of the roof rock movement during the simulated mining of 25 to 150 m (actual excavation of 50 to 300 mm in the model) in a single working face. Apparently, as the length of mining within 150 m, the direct roof falls as it is mined. But there is no obvious deformation damage to the basic roof and the overlying strata. The basic roof constitutes a fixed beam across both ends of the open cut and the mining coal wall.

**Figure 21:**
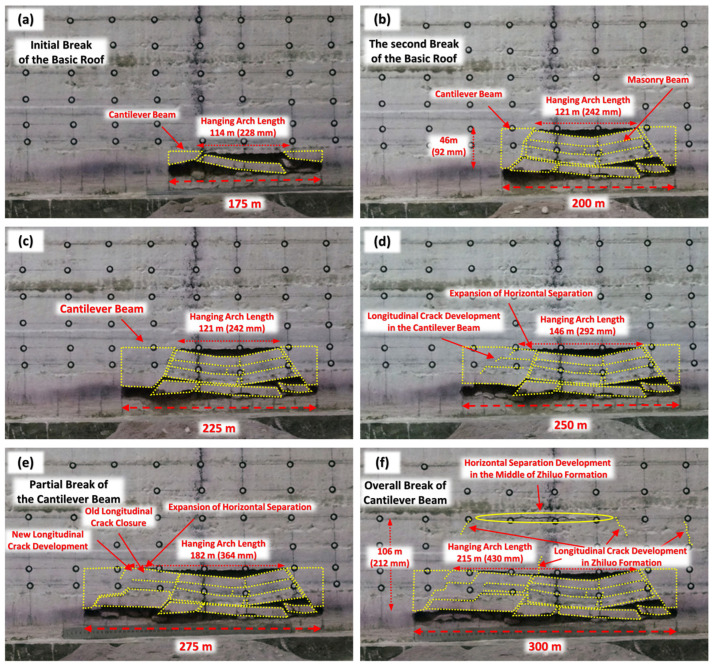
Overburden movement under simulated mining of 175 to 300 m widths. (**a**) 175 m. (**b**) 200 m. (**c**) 225m. (**d**) 250m. (**e**) 275 m. (**f**) 300 m.

Figure 21 shows the process of the roof rock movement during the simulated mining of 175 m to 300 m (actual excavation of 350–600 mm in the model) in a single working face. Within this mining length, intense deformation and destruction of the basic top occurs. Moreover, along with the increase in the mining length, obvious cracks and separations also appear in the upper Zhiluo Formation sandstone stratum.

From Figure 21a, it can be seen that when the mining length reached 175 m, the initial breakage of the lower layer in the basic roof occurred. An overhanging roof spacing of 114 m wide was formed above the initial break in the formation, and cantilever beam structure with almost equal width was formed on both sides of the extraction area.

As can be seen from Figure 21b, when the mining length was 200 m, the whole Jurassic Yan’an Formation above the coal seam occurred the initial breakage. The stratum in the broken area formed a typical masonry beam structure. A hypoconvex bowed horizontal separation, which is 121 m wide, was formed between the masonry beam structure and the upper Zhiluo Formation. Both sides of the masonry beam structure constituted the cantilever beam structure with a vertical height of 46 m along the broken line.

As shown in Figure 21c, there was no obvious change when the mining length reached 225 m. Only the width of the cantilever beam in front of the working face increased to a certain extent.

It can be seen from Figure 21d that when the mining length was 250 m, longitudinal cracks started to appear inside the cantilever beams in front of the working face, extending obliquely from the middle of the upper surface to the bottom. These longitudinal cracks, however, did not completely penetrate the entire cantilever beam structure. On the one hand, it indicates that the internal stress state of the cantilever beam structure continuously increased as the cantilever beam overhang distance became larger. When the internal stress exceeds the load-bearing limit, it leads to crack expansion and eventual breakage. On the other hand, the masonry beam structure was shown to have some supporting effect on the cantilever beams on both sides, which attenuated the degree of deformation and damage of the cantilever beams. In addition, the separation width between the basic roof and the Zhiluo Formation increased to 146 m along the mining direction.

When the mining length reached 275 m, the damage extent of the cantilever beam in front of the workings enlarged as shown in Figure 21e. But the evolution process was not simply characterized by crack size expansion and increased number of cracks. Instead, the longitudinal cracks formed in the previous stage were closed, while new cracks appeared in front. These new cracks remained unperforated throughout the cantilever beam structure. And the width of the separation between the basic roof and the Zhiluo Formation enlarged to 182 m.

As shown in Figure 21f, the cantilever beam structure in front of the workings was completely broken due to crack penetration when the mining length became 300 m. The width of the separation between the basic roof and the Zhiluo Formation enlarged to 215 m. The shape of this separation changed from the initial bow-shaped symmetric structure to a spoon-shaped bias structure. Moreover, the space behind the working surface was relatively large. Horizontal separation also appeared in the middle of the Zhiluo Formation. Vertical cracks developed on both sides of this separation, at the mid-position of the overhanging top, and near the open cut in the working face directly above. It indicates that the overall damage height was already increased upwards to 106 m at this stage. The height of the water flowing fractured zone measured in the field is about 21 times the mining height (116 m). The simulation results are consistent with the measured results. Zhiluo Formation began to be affected by mining disturbances. The whole goaf also began to form typical “three-zone” distribution characteristics of lower caving, middle fissure, and upper bending.

(2) Continuous Mining of Adjacent Multi-working Faces

**Figure 22:**
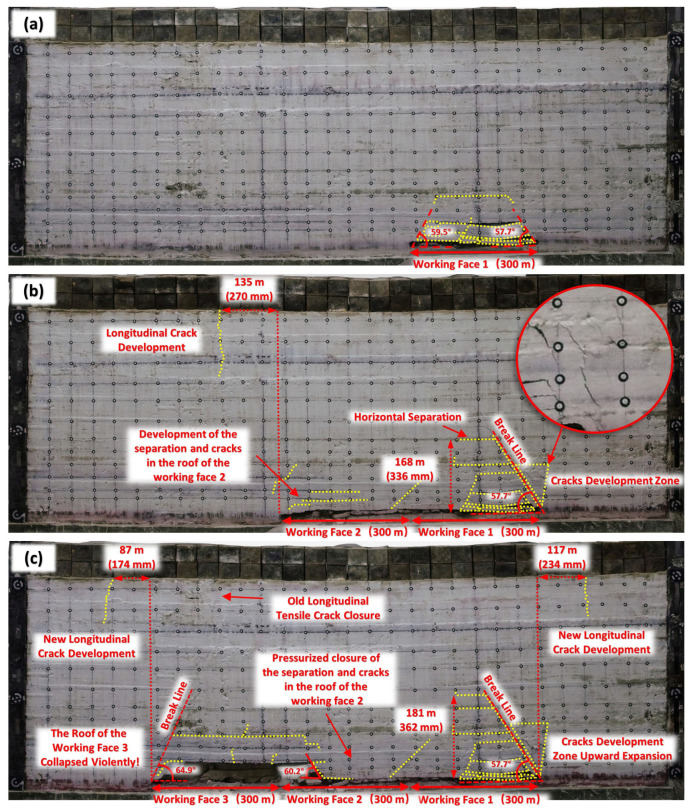
Overburden movement under simulated continuous mining of three working faces. (**a**) one working face. (**b**) two working faces. (**c**) three working faces.

According to the modeling scheme, the experimental results of the simulated mining width reaching 600 mm can also be used to analyze the strata movement characteristics on the inclined profile under the infinite length of the working face with 300 m wide. It has been described in detail previously. Additionally, the damage area of the overburden at this stage was surrounded by the top separation and the break lines on both sides. And the angles between the fracture line and the horizontal line on both sides (often referred to as the fracture angle) are 59.5° and 57.7°, respectively, as shown in Figure 22a.

Figure 22b shows the damage behavior of the overburden after the second 300 m wide working face was simulated to be mined. As the extraction space enlarged, the range of overburden movement expanded. The damage degree of overlying strata above the first working face was obviously increased. It indicated that the damage to the overburden is characterized by a cumulative effect. The fracture line on the right side extended upwards to a height of 168 m, but the fracture angle remained at 57.7°. Several horizontal departures occur near the left side of this break line. And an inverted triangle crack development zone with the break line as an oblique edge appeared in the right Zhiluo Formation. The damage degree of overlying strata above Working Face 2 was relatively slight. Only separation and vertical cracks not exceeding the height of Zhiluo Formation sandstone layer appear in the basic roof of the low level. There existed a break line across the sandstone layers of the Zhiluo Formation between the two working faces. This break line and the vertical crack on the left side of Working Face 2 constituted the broken rock mass of the Zhiluo Formation. And the broken rock mass formed a giant masonry beam structure with the broken area of Working Face 1. It should be noted that a longitudinal crack developed from top to bottom in the Zhidan Formation at 135 m away from the left side of Working Face 2. It indicates that when the total width of mining reaches 600 m, the high-level weakly cemented sandstone stratum began to be affected by mining, exhibiting a form of tension fracture.

Figure 22c shows the damage behavior of the overburden after the last working face was mined. The basic roof above Working Face 3 was severely damaged and broken into blocks of different sizes. Significant break lines appeared on the both left and right sides of Working Face 3. The fracture angle was 64.9° on the left side and 60.2° on the right side. The separation and cracks in the overlying strata above Working Face 2 were closed. This phenomenon indicates that the overburden rock of Working Face 2 already contacted the coal seam floor and acted as a support for the upper strata. The movement characteristics in the overlying strata above Working Face 1 changed insignificantly, while the extent of the crack development zone located to the right of Working Face 1 broadened to a certain extent. The total height of overburden damage increased slightly to 181 m. The longitudinal tensile crack that appeared in the Zhidan Formation in the previous phase was closed. However, new longitudinal tension cracks appeared at 87 m to the left of Working Face 3 and 117 m to the right of Working Face 1. It indicated that the upper part of the higher Zhidan formation would be repeatedly subjected to tensile and compressive deformation under the expanding area of exploitation.

### 4.2. Mechanical Mechanisms Controlling Key-Stratum Failure

According to the similar material simulation results in the previous section, the overburden rocks in the study area are deformed and damaged to different degrees under different mining widths. Mechanical bearing structures such as fixed beam and masonry beam and so on were formed in the near-field Jurassic Yan’an Formation, the mid-field Jurassic Zhiluo Formation, and the far-field Cretaceous Zhidan Formation. These mechanical bearing structures are the carriers of the displacement field, stress field, and overburden failure field from the underground goaf up to the surface. And the breakup of these structures can cause violent movement of the overburden stratum and the ground surface. In this section, the breakage mechanism of the overburden structure in each stratum is analyzed with the relevant theories.

#### 4.2.1. Failure Mechanism of Near-Field Jurassic Yan’an Formation

The basic roof of the middle-hard Jurassic Yan’an Formation, which is about 6 m away from the roof of the mined seam, is transformed from a fixed beam structure to a masonry beam structure in the middle of two cantilever beams on both sides at the early stage of the mining process with the increase in the excavation space. The structural transformation is determined by the initial breaking of the basic roof. Therefore, this section analyzes the breaking mechanism based on the traditional beam theory.

Equation (9) is the traditional beam theory to calculate the limit span of the fixed beam at tension break.

(9) $$L_{T}=h\sqrt{\frac{2R_{T}}{q}}$$

where $R_{T}$ is the tensile strength of the formation, h is the thickness of the formation, and q is the load applied.

The load on the consolidated basic roof is considered only its self-weight, and it is calculated by Equation (10)

(10) $$q_{1}=\gamma_{1}h_{1}$$

where $\gamma_{1}$ is the volume weight of the basic roof.

In the model of this study, the basic roof is simplified to two strata each with a thickness of 20 m. To facilitate the analysis, the two strata are further combined into an overall layer, so the total thickness is 40 m. From a safety point of view, the volume weight is $\gamma_{1}=2.45\times 10^{4}\;N/m^{3}$. If the tensile strength is taken as $\sigma_{T}=2.90\;MPa$, the tensile break limit span calculated by Equation (11) and Equation (12) is 97.3 m.

The above calculated data are close to the result of the simulation experiment (121 m). Considering the breaking angle (57.7°) on both sides, it can be deduced that the theoretical maximum mining width of the working face is 155.5 m when the basic roof initially breakage occurs. The value is less than 200 m in the similar material simulation, which is caused by ignoring the width of the cantilever beam on both sides of the breaking area. Therefore, it can be assumed that the initial breakage span of the basic roof obtained from experiments is consistent with the theoretically calculated value.

In short, under the geological conditions of the study area, when the working face is fully mined along the strike and the mining length is in the range of 155.5 to 200.0 m, the near-field Jurassic Yan’an Formation, which is about 6 m away from the roof of the mined seam, breaks.

#### 4.2.2. Failure Mechanism of Mid-Field Jurassic Zhiluo Formation

The Zhiluo Formation sandstone in the overburden, 46 m from the mined coal seam, has a large thickness (120 m) and a medium-hard lithology. Thus, this formation has a controlling effect on the upper Jurassic Anding Formation. In this subsection, the breaking mechanism is analyzed based on the key stratum theory.

First, we determine whether the Zhiluo Formation is an inferior key stratum. Table 5 shows the calculated physico-mechanical parameters of the Jurassic Zhiluo Formation, the Anding Formation, and the Cretaceous Zhidan Formation.

The self-weight load of the fine sandstone strata of the Jurassic Zhiluo Formation is:

(11) $$q_{21}=\gamma_{21}h_{21}=2904.0\;\mathrm{kPa}$$

The loads considering the influence of the upper k layers over the lower key stratum [33] are given in Equation (12):

(12) $$q=\frac{E_{1}h_{1}^{3}\sum_{i=1}^{k}\gamma_{i}h_{i}}{\sum_{i=1}^{k}E_{i}h_{i}^{3}}$$

The calculated loads of the second to fourth strata are 3742.4 kPa, 4605.2 kPa, and 5342.8 kPa, respectively. The uppermost thick and weakly cemented Cretaceous Zhidan Formation is subjected to a load of 813.8 kPa. Obviously, $q_{21}<q_{22}<q_{23}<q_{24}$, and $q_{21}>q_{25}$. Therefore, the Jurassic Zhiluo Formation can be judged as an inferior key stratum. Taking $q_{24}$ and the tensile strength $\sigma_{T}=3.00\;MPa$ of the Zhiluo Formation into Equation (11). The theoretical value of the limit span (127.2 m) of the mid-field Jurassic Zhiluo Formation can be obtained. Considering the breaking angle (57.7°), it can be deduced that the maximum mining width of the working face is 387.2 m when the Zhiluo Formation is broken in theory. This value is between the single working face mining width (300 m) and the total width (600 m) of adjacent double working faces in the simulation experiment. Therefore, it can be considered that the limit span of inferior key stratum obtained by experiment is consistent with the theoretical calculation value.

In short, when the mining length is in the range of 387.2 to 600.0 m, the mid-field Jurassic Zhiluo Formation, which is about 46 m away from the roof of the mined seam, breaks.

#### 4.2.3. Deep-Beam Failure Mechanism of Far-Field Cretaceous Zhidan Formation

In the previous two subsections, the mechanical theory used in the analysis of the fracture mechanism of the near-field and mid-field rock structure is essentially a long beam (Euler–Bernoulli beam) structural mechanics analysis method that ignores the influence of internal shear strain. However, the long beam theory is only applicable when the ratio of rock thickness to span is within 1/4. When the thickness–span ratio of strata exceeds 1/4, the influence of shear strain inside on deformation cannot be ignored. There is a large error in the theoretical calculation value. In this case, the deep beam (Timoshenko beam) structural mechanics analysis method should be used to solve the problem [36].

In this study, the thicknesses of the near-field Jurassic Yan’an Formation, the mid-field Jurassic Zhiluo Formation, and the far-field Cretaceous Zhidan Formation are 40 m, 120 m, and 340 m, respectively. In the simulation experiment, the three formations were broken at mining widths of 200 m, 600 m, and 900 m. Considering the fracture angle of 57.7°, the maximum thickness–span ratios of the three formation before breakage can be calculated as 0.21, 0.23, and 0.78. It is clear that the maximum thickness–span ratio of the near-field and the mid-field formations are within 1/4, which meets the requirements of the long beam structural mechanics. The thickness–span ratio of the far-field formations is far more than 1/4, which no longer meets the requirements of long beam structure mechanics. Therefore, this subsection uses the deep beam theory to analyze the breakup mechanism of the far-field Cretaceous Zhidan Formation. Due to the existence of aeolian sand layer with a thickness of 100 m at the top of the overburden in this study, it can be considered that the Zhidan Formation is in a fixed boundary condition at both ends.

**Figure 23:**
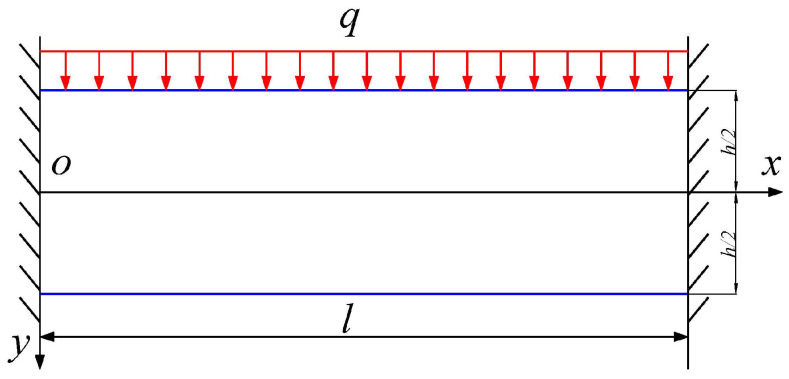
Mechanical model of deep beam fixed at both ends.

Based on the semi-inverse method in elastic mechanics, the biharmonic stress function of the deep beam structure fixed at both ends in Figure 23 can be assumed to be the following quintic polynomial:

(13) $$\Phi =A\left(\frac{y^{5}}{5}-x^{2}y^{3}\right)+Bxy^{3}+Cy^{3}+Dy^{2}+Fx^{2}y+Gxy+Hx^{2}$$

In Equation (13), *A*, *B*,…, *H* are seven undetermined coefficients determined by boundary conditions.

The self-gravity of the deep beam increases with its height. However, when the self-gravity increases to a certain extent, it affects the internal stress distribution. It is still difficult to solve for the self-gravity stresses in deep beam structures. In this study, the self-gravity of the far-field Cretaceous Zhidan Formation and the load of the overlying 100 m thick aeolian sand are combined into the overlying uniform load. This treatment is not only consistent with the actual situation of the project, but also greatly simplifies the mechanical analysis.

After the above simplified treatment, it can be seen that the stress components in the deep beam structure under the condition of no volume force are:

(14) $$\sigma_{x}=\frac{\partial^{2}\Phi }{\partial y^{2}},\sigma_{y}=\frac{\partial^{2}\Phi }{\partial x^{2}},\tau_{xy}=\frac{\partial^{2}\Phi }{\partial x\partial y}$$

Bringing Equation (14) into Equation (13) can get:

(15) $$\begin{array}{cc} \sigma_{x} & =2A\left(2y^{3}-3x^{2}y\right)+6Bxy+6Cy+2D\\ \sigma_{y} & =-2Ay^{3}+2Fy+2H\sigma_{y}\\ \tau_{xy} & =6Axy^{2}-3By^{2}-2Fx-G \end{array}$$

The boundary conditions of the deep beam structure fixed at both ends are:

(16) $$\begin{array}{cc} y & =-\frac{h}{2},\sigma_{y}=-q,\tau_{xy}=0\\ y & =\frac{h}{2},\sigma_{y}=0,\tau_{xy}=0 \end{array}$$

Combining Equations (14)–(16), the expressions of stress components in deep beam with clamped boundary conditions at both ends are obtained:

(17) $$\begin{array}{c} \sigma_{x}=\frac{4q}{h^{3}}y^{3}-\frac{6q}{h^{3}}x^{2}y+\frac{6ql}{h^{3}}xy-\frac{q\left(l^{2}+h^{2}-\upsilon h^{2}\right)}{h^{3}}-\frac{\upsilon q}{2}\\ \sigma_{y}=-\frac{2q}{h^{3}}y^{3}-\frac{3q}{2h}y-\frac{q}{2}\\ \tau_{xy}=\frac{6q}{h^{3}}{xy}^{2}-\frac{3ql}{h^{3}}y^{2}-\frac{3q}{2h}x+\frac{3ql}{4h} \end{array}$$

In Equation (17), $\sigma_{x}$ is the horizontal stress at any point in the deep beam; $\sigma_{y}$ is the vertical stress; and $\tau_{xy}$ is the shear stress; q is the load on the deep beam; υ Poisson ratio; h is the thickness of the deep beam; and l is the span of the deep beam.

(1) Tensile Failure

The transformation relationship between the maximum principal stress $\sigma_{1}$ and the horizontal, vertical, and shear stress components is:

(18) $$\sigma_{1}=\frac{\sigma_{x}+\sigma_{y}}{2}+\sqrt{\frac{{\left(\sigma_{x}-\sigma_{y}\right)}^{2}}{4}+\tau_{xy}^{2}}$$

**Figure 24:**
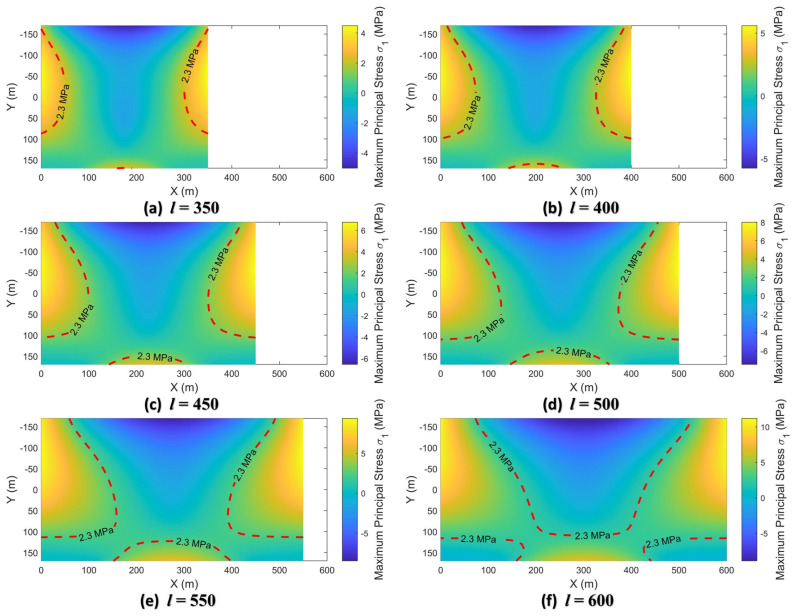
Distribution of maximum principal stresses in deep beams with different spans.

The maximum principal stress at any point inside the deep beam is calculated by bringing each stress component in Equation (17) into Equation (18). In this study, the thickness $h_{3}$ of the far-field Cretaceous Zhidan Formation is 340 m and the Poisson ratio $\upsilon_{3}$ is 0.3. The load $q_{3}$ of the Zhidan group rock stratum, including the self-weight load and the uniform load $q_{30}$ applied by the overlying aeolian sand layer, is 8.91 MPa. After substituting the above parameters, the maximum principal stress $\sigma_{1}$ can be further expressed as a function expression related only to coordinates (*x*, *y*) and deep beam span *l*. In this study, the spatial distribution of the maximum principal stress was calculated when the span *l* is 350 m, 400 m, 450 m, …, 600 m, respectively, as shown in Figure 24.

The characteristics of the maximum principal stress distribution can be found from Figure 24 as follows.

The maximum principal stresses, including tensile stresses with positive values and compressive stresses with negative values, increase in magnitude as the span *l* of the Zhidan Formation increases. Moreover, the increment of the maximum principal tensile stress is significantly larger than that of the maximum principal compressive stress.

The internal tensile stress concentration areas are located at the upper side of both ends and the middle area of the bottom of the deep beam. The compressive stress concentration area is located in the middle area of the top of the deep beam, showing a “funnel” shape. In addition, there are regions where the maximum principal stresses are close to zero at the bottom ends of the deep beams.

The tensile strength $\sigma_{T}=2.30\;MPa$ of the Zhidan Formation can be used to further obtain the tensile failure range in deep beams with different spans, as shown in part of the area where the maximum principal stress value exceeds the red dotted line in Figure 24. When the span *l* is in the range of 350 m to 600 m, the tensile damage zones appear inside the deep beam. As *l* increases, the tensile failure zone expands from the top and bottom intermediate regions at the ends of the deep beams to the interior. When the span *l* reaches 600 m, the tensile failure zone of the two regions at the top and bottom of the deep beam is connected, indicating that the stratum experiences complete tensile failure.

(2) Shear Failure

The transformation relationship between the maximum shear stress $\tau_{max}$ and the horizontal, vertical, and shear stress components is:

(19) $$\tau_{max}=\sqrt{\frac{{\left(\sigma_{x}-\sigma_{y}\right)}^{2}}{4}+\tau_{xy}^{2}}$$

**Figure 25:**
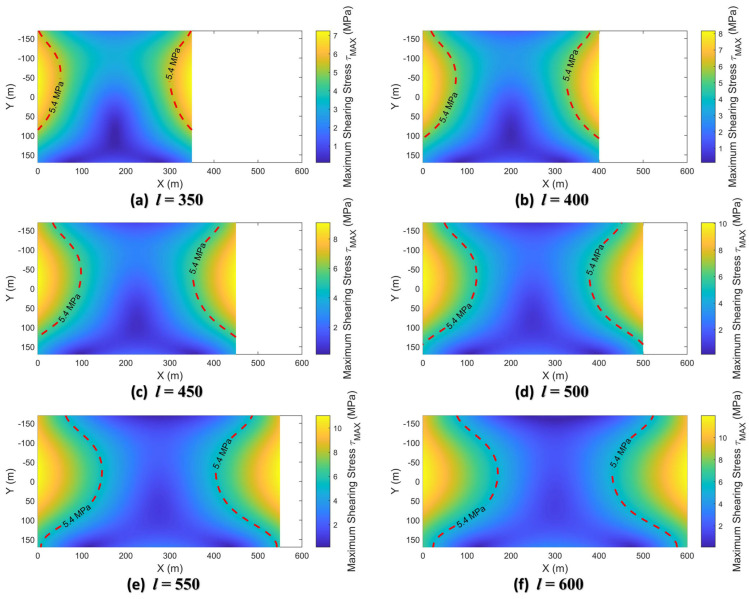
Distribution of maximum shear stresses in deep beams with different spans.

The maximum shear stress at any point inside the deep beam is calculated by bringing each stress component in Equation (17) into Equation (19). After substituting the parameters, the maximum shear stress $\tau_{max}$ can be further expressed as a function expression related only to coordinates (*x*, *y*) and deep beam span *l*. In this study, the spatial distribution of the maximum shear stress was also calculated when the span *l* is 350 m, 400 m, 450 m, …, 600 m, respectively, as shown in Figure 25.

The characteristics of the maximum shear stress distribution can be found from Figure 25 as follows.

The maximum shear stresses magnitude increase as the span *l* of the Zhidan Formation increases.

In the middle of the deep beam, a “dumbbell”-shaped low-shear-stress zone is formed. The bottom area of the low-shear-stress zone is larger than the top. The shear stress concentration areas are located on both sides of the deep beam. As the span *l* increases, the range of shear stress concentration gradually expands from both sides to the middle and lower parts.

The shear strength of rock material is generally between its compressive strength and tensile strength. In the case of effective stress, the total shear strength is deducted from the friction strength to obtain the cohesion. From another point of view, cohesion can be regarded as the shear strength of the failure surface without any normal stress. Therefore, we continue to use the cohesion C=5.40 MPa of the Zhidan Formation in the previous simulation experiment and approximately frame the shear failure range of deep beams with different spans, as shown in part of the area where the maximum shear stress value exceeds the red dotted line in Figure 25. When the span *l* is in the range of 350 m to 600 m, the shear damage zones appear inside the deep beam. As *l* increases, the tensile failure zone expands from both ends of the deep beam to the middle and bottom regions. When the span *l* reaches 550 m, the shear failure zone at both ends of the deep beam penetrates up and down., indicating that the stratum is completely shear failure.

(3) Overlapping Destruction of Tension and Shear

**Figure 26:**
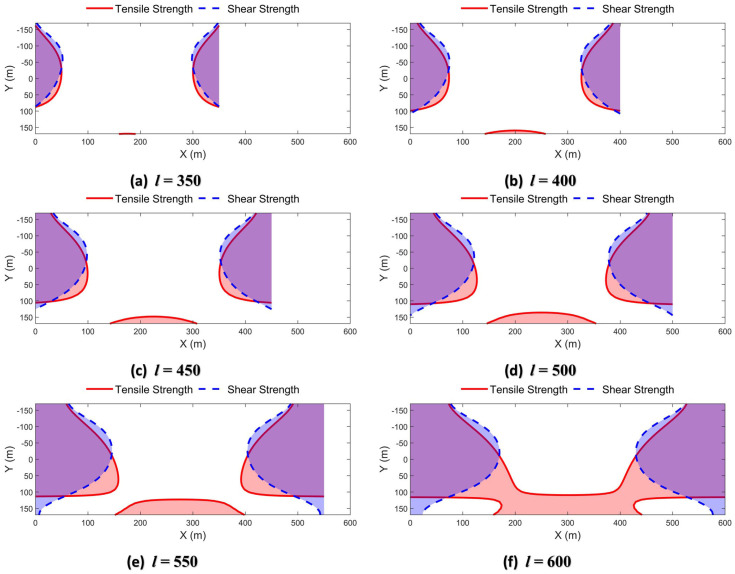
Distribution of tensile–shear superimposed failure in deep beams with different spans.

The above analysis shows the complex characteristics of the failure mode of the deep beam structure. In order to give relatively accurate span *l* corresponding to the complete failure of the Zhidan Formation and the working face width *L*, the tensile and shear destruction zones in Figure 24 and Figure 25 are superimposed here. The superposition results are shown in Figure 26. From Figure 26, the tension–shear damage characteristics of deep beam structures are as follows.

The upper part of both sides of the deep beam is consistently subjected to the superimposed effects of tensile and shear failure. With the increase in span *l*, the tensile–shear superimposed failure zone in front of the mining is continuously transferred to the front of the mining. This is consistent with the simulation experiment result that the old longitudinal crack at the top of the Zhidan Formation is closed and new longitudinal crack appears in front of the working face.

The middle area at the bottom of the deep beam is only affected by tensile failure. It theoretically explains the occurrence of several separations and cracks in the middle region at the bottom of the giant thick Zhidan Formation in the previous simulation.

**Figure 27:**
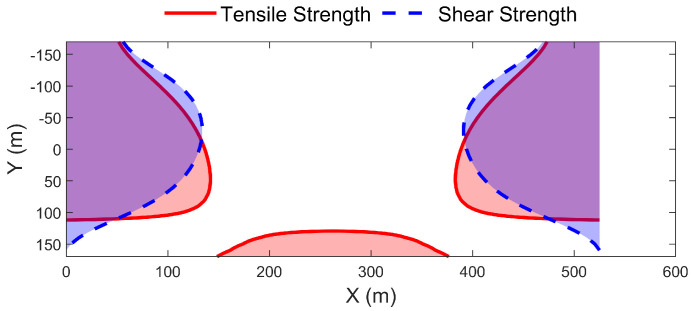
Distribution of tensile–shear superimposed failure in deep beams with span *l* is 525 m.

When the span *l* reaches 550 m, the shear damage zone penetrates through both ends of the deep beam first. It means that the corresponding damage span *l* of Zhidan group penetrating damage should be in the range of 500 m to 550 m. Through a number of trial calculations, eventually a relatively accurate thorough shear span *l* is obtained for 525 m. The distribution of the corresponding tension–shear damage zones is shown in Figure 27. Considering the breaking angle (57.7°), the limit mining width *L* when the far-field Cretaceous Zhidan Formation is completely sheared off is 988.7 m, which is slightly larger than the total width of the three working faces in the simulation experiment.

In Figure 26f, when the span *l* reaches 600 m, the tensile failure zone of the upper and lower middle regions at both ends is completely connected. It shows that the damage degree of deep beam structure will further increase after shearing off.

In short, when the mining length is in the range of 900.0 to 988.7 m, the far-field Cretaceous Zhidan Formation, which is about 296 m away from the roof of the mined seam, occurs, penetrating failure dominated by shear breakage. Subsequently, the tensile and shear failure areas are further increased until the deep beam structure is completely broken.

## 5. Conclusions

This study establishes an integrated observation–experiment–model framework to advance the understanding and prediction of sudden subsidence in deep mining areas characterized by high-positioned, ultra-thick, and weakly cemented key strata. The major conclusions are as follows:

(1) A refined 2D SBAS-InSAR technique was developed to overcome the intrinsic geometric limitations of traditional 1D InSAR. By quantitatively analyzing the systematic errors induced by north–south and east–west horizontal displacements, we proposed a two-direction decomposition strategy using Sentinel-1A adjacent orbits. The method effectively reduces the three-dimensional monitoring error from approximately 50 mm to below 20 mm. This not only enables accurate reconstruction of the true vertical displacement field but also allows reliable extraction of the subsidence basin boundary angles in strike (52.3°–58.6°) and dip directions (44.3°–48.2°) under complex mining conditions.

(2) Large-scale physical similarity simulations vividly reproduced the complete overburden failure process, revealing the sequential rupture behaviors of multi-level key strata. The experiments demonstrated that, as mining width increases, the near-field Yan’an Formation transitions from fixed-beam to masonry-beam structures, the mid-field Zhiluo Formation undergoes inferior key-stratum breaking with cumulative deformation effects, and the far-field Zhidan Formation exhibits deep-beam tensile–shear coupled failure. These observations provide indispensable visual evidence for understanding the nonlinear evolution of subsurface mechanical structures.

(3) A theoretical mechanical model was established to quantify the critical conditions governing key-stratum failure, bridging surface deformation with deep-rock structural mechanics. By integrating InSAR-derived deformation fields with experimental insights, the critical width-to-depth ratios triggering breakage of the Yan’an (0.21–0.27), Zhiluo (0.53–0.82), and Zhidan Formations (1.22–1.34) were determined. The deep-beam analytical model further explains the dominance of shear penetration in the ultimate failure of the ultra-thick Zhidan Formation, which controls the occurrence of sudden, large-scale subsidence.

(4) The study shifts the field from describing surface deformation to elucidating subsidence mechanisms and establishing prediction-oriented indicators. The proposed monitoring–simulation–mechanics integrated framework provides a principled path for constructing early-warning models for abrupt subsidence. It offers generalizable thresholds, mechanical insights, and monitoring strategies applicable to mining regions with similar stratigraphic and geological conditions worldwide.

Overall, this research contributes a comprehensive theoretical and technical foundation for proactive subsidence risk assessment. It significantly enhances the capability to detect, explain, and ultimately predict catastrophic ground collapse, promoting safer and more sustainable underground mining practices on a global scale.

## Figures and Tables

**Table 1:** The main parameters of the Sentinel-1 A satellite SAR images used.

Category	Parameter
Acquisition Date Range	2017.03.10–2022.06.05
Azimuth Angle	81.8°
Incident Angle	44° (West, Path84), 38° (East, Path11)
Polarization Mode	VV
Wave Band	C
Wave Length	5.56 cm
Imaging Mode	Interferometric Wide sath (IW)
Data Format	Single Look Complex (SLC)

**Table 2:** The segmentation processing information of SBAS-InSAR.

Time Range No.	Western SAR Data Set Time Range	Eastern SAR Data Set Time Range	Mining Status of the Working Face
1	20170317–20180604	20170312–20180611	2217: 0–1160 m 2101: 0–1160 m 2215: 0 m 2201: 0 m
2	20180604–20190729	20180611–20190724	2217: 1160–2600 m 2101: 1030–2080 m 2215: 0 m 2201: 0 m
3	20190729–20210519	20190724–20210514	2217: 2600 m 2101: 2080 m 2215: 0–1330 m 2201: 0–1020 m
4	20210811–20220526	20210806–20220602	2217: 0–2600 m 2101: 0–2080 m 2215: 1330 m 2201: 1020 m

**Table 3:** Physical and mechanical parameters of stratigraphic prototype and model strata.

No.	Strata Lithologic	Stratigraphic Prototype				Similarity Model			
		Thicknesses, (m)	ρ $\left(kg/m^{3}\right)$	$\sigma_{C}$ $\left(MPa\right)$	$\sigma_{m}$ $\left(MPa\right)$	Thicknesses, (m)	ρ $\left(kg/m^{3}\right)$	$\sigma_{C}$ $\left(MPa\right)$	$\sigma_{m}$ $\left(MPa\right)$
1	Zhidan Sandstone	185	2150	14.44	2.30	37	1500	18.1	2.9
2	Sandy Mudstone	40	2350	32.93	2.80	8	1500	41.2	3.5
3	Fine Sandstone	50	2300	24.70	3.00	10	1500	30.9	3.8
4	Sandy Mudstone	40	2350	30.15	2.80	8	1500	37.7	3.5
5	Zhiluo Sandstone	120	2420	32.43	3.00	24	1550	40.5	3.8
6	Sandy Mudstone	20	2450	30.29	2.80	4	1550	37.9	3.5
7	Medium-grained Sandstone	20	2400	31.43	2.90	4	1550	39.3	3.6
8	Sandy Mudstone	6	2480	38.33	2.50	1.2	1550	47.9	3.1
9	#2-2 Coal Seam	6	1350	11.44	1.35	1.2	900	14.3	1.7
10	Sandy Mudstone	6	2500	35.22	2.80	1.2	1550	44.0	3.5
11	Coal Seam Floor	20	2450	41.45	3.20	4	1550	51.8	4.0

**Table 4:** Weight Ratio of Similar Materials.

No.	Strata Lithologic	Stratigraphic Prototype	
		Overall Material Ratio Yellow Sand: Mica Powder: Cementation	Cementation Ratio Gesso: Calcium Carbonate
1	Zhidan Sandstone	80:19:1	5:5
2	Sandy Mudstone	80:18:2	3:7
3	Fine Sandstone	76:21:3	7:3
4	Sandy Mudstone	80:18:2	5:5
5	Zhiluo Sandstone	78:18:4	7:3
6	Sandy Mudstone	75:22:3	5:5
7	Medium-grained Sandstone	73:23:4	7:3
8	Sandy Mudstone	75:21:4	5:5
9	#2-2 Coal Seam	80:17:3	3:7
10	Sandy Mudstone	75:21:4	7:3
11	Coal Seam Floor	73:23:4	5:5

**Table 5:** Physical and mechanical parameters of the formation used for key stratum determination.

No.	Strata Lithologic	Thicknesses (m)	Volume Weight ($10^{4}\;N/m^{3}$)	Elastic Modulus (MPa)	Tensile Strength (MPa)
25	Zhidan Sandstone	340	2.15	8.00	2.30
24	Sandy Mudstone	40	2.35	8.50	2.80
23	Fine Sandstone	50	2.30	9.50	3.00
22	Sandy Mudstone	40	2.35	8.80	2.80
21	Fine Sandstone	120	2.42	12.00	3.00

## Data Availability

The original contributions presented in this study are included in the article. Further inquiries can be directed to the corresponding author.

## Abbreviations

The following abbreviations are used in this manuscript:

InSAR	Interferometric Synthetic Aperture Radar
SBAS	Small Baseline Subset
ESA	European Space Agency
LOS	Line of Sight
ASD	Actual Surface Displacement
VD	Vertical Displacement
